# SlMYB72 and SlMYB75 antagonistically regulate trichome formation *via* the MYB-bHLH-WD40 complex in tomato

**DOI:** 10.1016/j.jbc.2025.108313

**Published:** 2025-02-13

**Authors:** Tiancheng Qi, Mengbo Wu, Sijie Wang, Ying Yuan, Xin Xu, Qiongdan Zhang, Yongfei Jian, Dan Qiu, Yulin Cheng, Baowen Huang, Zhengguo Li, Weiqing Zhang, Wei Deng

**Affiliations:** 1 Key Laboratory of Plant Hormones and Development Regulation of Chongqing, School of Life Sciences, Chongqing University, Chongqing, China; 2 Key Laboratory of Bio-Resource and Eco-Environment of Ministry of Education, College of Life Sciences, Sichuan University, Chengdu, Sichuan, China

**Keywords:** multicellular trichome, MYB-bHLH-WD40 complex, tomato, transcriptional regulation, trichome formation

## Abstract

Trichomes are specialized epidermal outgrowths serving as protective barriers for plants against various stresses such as herbivore attacks. MYB-bHLH-WD40 complex is of great significance for unicellular trichome formation in *Arabidopsis*, whereas its role in the formation of multicellular trichomes in tomatoes remains largely unknown. In the present study, we identified that the R2R3-type MYB transcription factor SlMYB72 promotes the formation of type II, V, and VI trichomes by inhibiting the expression of *SlCycB2*, a repressor of trichome initiation. SlMYB75 is a negative regulator of trichome formation and positively regulates *SlCycB2* expression. Interaction analyses showed that SlMYB72 and SlMYB75 can form MYB-bHLH-WD40 complexes with SlbHLH150 and SlTTG1, respectively, through mutual interactions. The dual-luciferase assay demonstrated that the regulatory functions of SlMYB72 and SlMYB75 in *SlCycB2* expression can be enhanced by their corresponding MYB-bHLH-WD40 complexes. Interestingly, yeast-three-hybrid assay indicated that SlMYB75 competes with SlMYB72 for SlbHLH150 and SlTTG1, and counterbalances the down-regulation of *SlCycB2* expression controlled by SlMYB72 alone, which is further confirmed by genetic hybrid experiments. These results reveal that SlMYB72 and SlMYB75 antagonistically regulate trichome formation and *SlCycB2* expression through MYB-bHLH-WD40 complexes. These findings provide a novel perspective and theoretical basis for the formation of multicellular trichomes in tomatoes and the development of highly resistant plants.

Sessile plants usually encounter complicated and variable environments, and they handle these challenges through various intricate mechanisms such as the formation of specialized tissue structures. Trichomes are specialized hair-like structures derived from epidermal cells that are widely distributed on the surface of leaves, stems, and flowers of plants and act as protective barriers against various biotic and abiotic stresses (1). They are unicellular or multicellular accessories that are distinct in size, shape, and structure and can be classified as glandular and non-glandular based on their metabolic characteristics (2). Glandular trichomes synthesize various metabolites such as terpenoids, polyphenols, acyl glycosides, and alkaloids. These metabolites not only provide plants with resistance against the invasion of pathogens and herbivores but are also crucial for their growth and development (3, 4, 5). Non-glandular trichomes generally serve as physical barriers to protect plants from extreme temperatures, ultraviolet radiation, and herbivore infestations, and they also contribute to water absorption, metal detoxification, and seed dispersal (6). Since trichomes are important for plant fitness and stress responses, the process of trichome formation is rigorously controlled by hormones, epigenetic modifications, and transcriptional regulation (1, 7).

*Arabidopsis thaliana* (hereafter *Arabidopsis*) possesses unicellular and non-glandular trichomes and serves as an ideal model for studying trichome development, with its transcriptional regulatory network well-established (8, 9). To date, several important proteins involved in *Arabidopsis* trichome formation have been identified, most of which are transcription factors, including myeloblastosis (MYB) transcription factors, basic helix-loop-helix (bHLH) transcription factors, homeodomain leucine zipper (HD-ZIP) proteins, and WD40-repeat proteins (10). Among these, the R2R3-type MYB transcription factor GLABROUS1 (GL1) was the first regulatory protein identified to positively mediate trichome formation (11). It can directly regulate the expression of *GLABRA2* (*GL2*) and *TRANSPARENT TESTA GLABRA2* (*TTG2*) (12, 13). The HD-ZIP protein GL2 is a common regulator that promotes trichome initiation (14) and can be specifically targeted by the bHLH transcription factors GLABRA3 (GL3) and ENHANCER of GL3 (EGL3) (15). GL3 and EGL3 function redundantly in the process of trichome formation, and another bHLH protein, TRANSPARENT TESTA8 (TT8), is also important for trichome development along leaf margins (16). GL3/EGL3 can interact with the WD40-repeat protein, TRANSPARENT TESTA GLABRA1 (TTG1), a multifunctional plant-specific protein widely present in angiosperms (17, 18). The absence of TTG1 results in the lack of trichomes in *Arabidopsis*, indicating a positive regulatory role of TTG1 in trichome initiation (17, 18). Besides interacting with GL3/EGL3, TTG1 can form the MYB-bHLH-WD40 (MBW) complex by interacting with GL1, which directly targets *GL2*/*TTG2* and initiates trichome development (19). Similar to GL1, several R2R3-MYBs (AtMYB23 and AtMYB82) also form MBW activator complexes with TTG1 and GL3/EGL3 to regulate *GL2/TTG2* expression, highlighting the core regulatory function of MBW in trichome formation in *Arabidopsis* (1). Notably, these MBW activator complexes can be disrupted by R3-type MYBs, such as TRIPTYCHON (TRY) and CAPRICE (CPC), which play an inhibitory role in trichome formation (2, 20). These R3-MYBs compete with GL1 for GL3/EGL3 to form a new complex consisting of R3-MYB, GL3/EGL3, and TTG1, thereby repressing the activation of *GL2*/*TTG2* (21). Collectively, MBW complexes play an important role in *Arabidopsis* trichome development, a process simultaneously regulated by numerous essential proteins and environmental factors (8). However, whether this regulatory model *via* the MBW complex applies to plants with multicellular trichomes, such as tobacco and tomato, has not yet been reported (5, 22).

The present study reveals that two R2R3-type MYBs, SlMYB72 and SlMYB75, exert opposite effects on the regulation of tomato trichome density and *SlCycB2* expression, which are further enhanced by their corresponding MBW complex with SlbHLH150 and SlTTG1. Additionally, SlMYB75 competes with SlMYB72 for binding to SlbHLH150 and SlTTG1. These findings demonstrate the roles of MBW complexes formed by SlMYB72 or SlMYB72, along with SlbHLH150 and SlTTG1, in the formation of tomato trichomes and the antagonistic regulatory relationship of these MBW complexes, providing a novel perspective on the regulatory mechanism of multicellular trichome formation.

## Results

### SlMYB72 positively regulates trichome formation in tomato leaves

**Figure 1:**
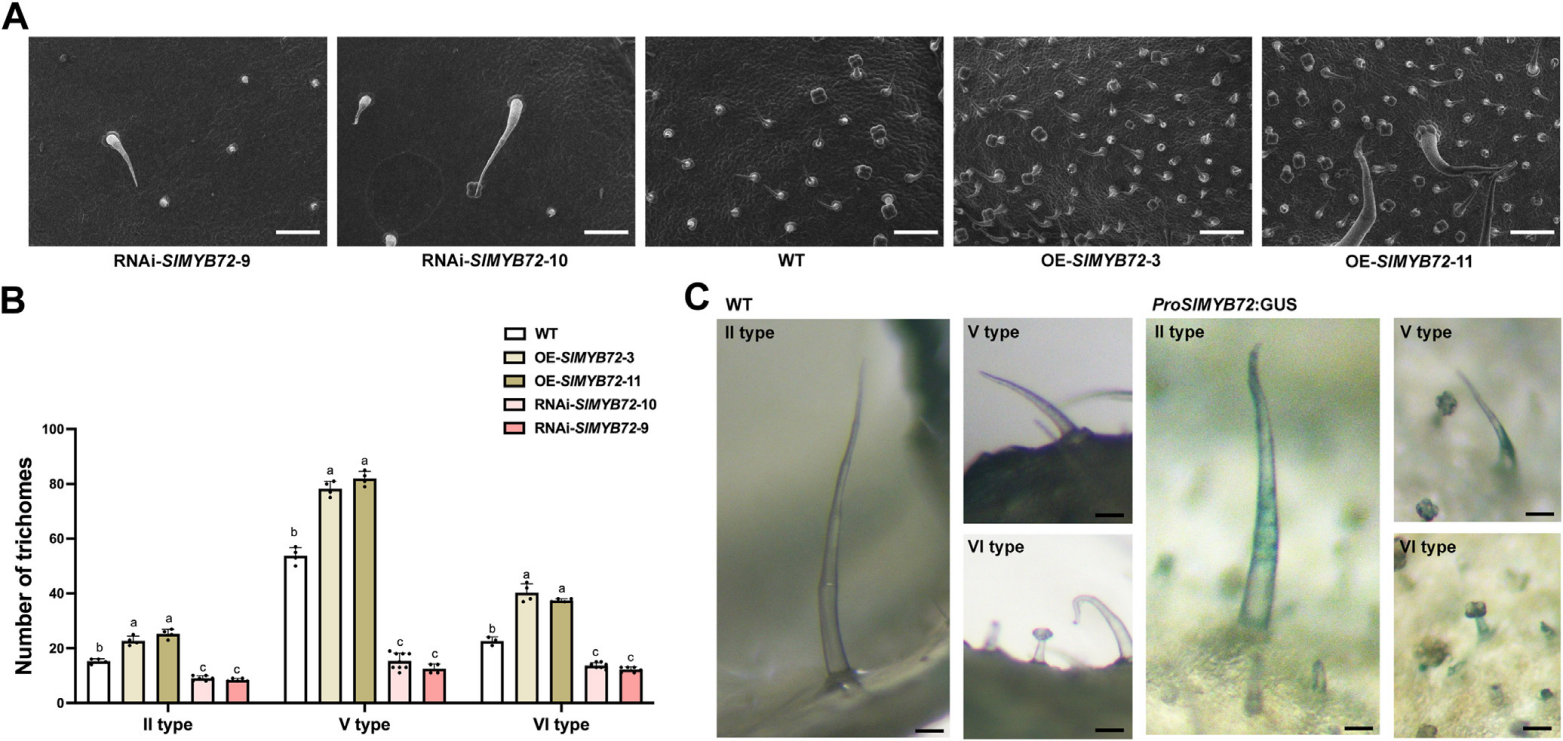
**SlMYB72 positively regulates the formation of type II, V, and VI trichomes.***A*, scanning electron micrographs of trichomes on the surface of tomato leaves of *SlMYB72* transgenic plants (RNAi-*SlMYB72* and OE-*SlMYB72*). Scale bars = 50 mm. *B*, trichome counting in RNAi-*SlMYB72* and OE-*SlMYB72* plants. The number of type II trichomes within a 0.5 cm^2^ region and types V and VI trichomes within a 2.2 mm^2^ region of the leaves were counted. One sector per leaf was dissected to examine the number of type II, V, and VI trichomes on the blade surface, and at least three leaves of each independent transgenic line were collected for trichome counting. Data were obtained from at least three biological replicates and presented as the mean ± standard deviation (SD). Distinct lowercase letters indicate statistically significant differences (*p* < 0.05). *C*, expression pattern of *SlMYB72* in type II, V, and VI trichomes, as estimated by GUS staining. The *GUS* reporter gene was controlled by the *SlMYB72* promoter. *Left*, GUS staining of WT; *Right*, GUS staining of *ProSlMYB72:GUS*. Scale bars = 0.25 mm. WT, wild type.

**Figure S1:**
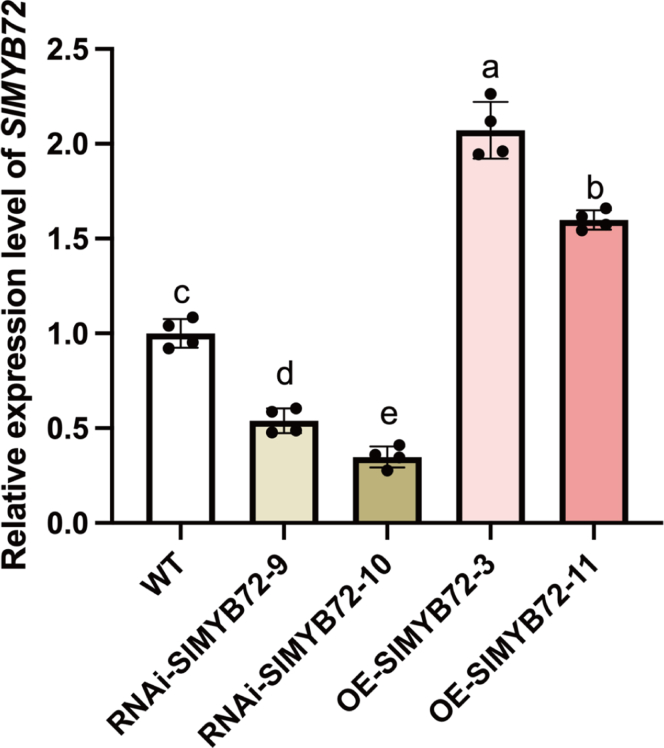
**Figure S1. RT-qPCR analysis of *SlMYB72* expression in RNAi-*SlMYB72* and OE- *SlMYB72* plants.** Data are presented as ± SD of at least three biological replicates.

SlMYB72 is a multifunctional R2R3-type MYB transcription factor that plays important roles in autophagy, pollen development, and the metabolism of chlorophyll, carotenoids, and flavonoids (23, 24). Given that flavonoid biosynthesis is closely related to trichome formation and several co-regulators involved in both pathways have been reported previously (25, 26, 27), we investigate whether SlMYB72, a regulator of flavonoid biosynthesis, also plays a role in trichome formation in tomatoes. Trichome density was analyzed using *SlMYB72* interference lines (RNAi-*SlMYB72*) and overexpression lines (OE-*SlMYB72*), which were controlled by *Cauliflower mosaic* virus (CaMV) 35S promoters and developed in our previous study (24). The expression of *SlMYB72* was actually decreased in the RNAi-*SlMYB72* plants, while it was elevated in the OE-*SlMYB72* lines, validating the reliability of the *SlMYB72* transgenic plants (Fig. S1). The density of trichomes on the leaves of the wild type (WT) and *SlMYB72* transgenic lines was determined using scanning electron microscopy. In comparison to WT ‘Micro-Tom’ tomato leaves, the trichome density on the leaves of RNAi-*SlMYB72* lines was significantly reduced, whereas OE-*SlMYB72* lines showed a notable increase in trichome density (Fig. 1*A*). Additionally, the number of type II, V, and VI trichomes on the leaves of the WT and *SlMYB72* transgenic plants was counted. The results revealed that overexpression of *SlMYB72* augmented, while knockdown of *SlMYB72* reduced, the abundance of type II, V, and VI trichomes (Fig. 1*B*). These results imply a positive correlation between trichome abundance and the expression level of *SlMYB72*.

To investigate the expression pattern of *SlMYB72* in trichomes, the *β-Glucuronidase* (*GUS*) reporter gene controlled by the *SlMYB72* promoter was expressed in the WT background, and the resulting plants were marked as *ProSlMYB72*:*GUS*. The results of the GUS staining analysis demonstrated that the *SlMYB72* promoter could drive the expression of the *GUS* reporter gene in type II, V, and VI trichomes, indicating that *SlMYB72* is expressed in these types of trichomes (Fig. 1*C*). These results confirm that SlMYB72 positively regulates the formation of type II, V, and VI trichomes in tomato leaves.

### SlMYB72 binds to the promoter of *SlCycB2* and represses its expression

**Figure 2:**
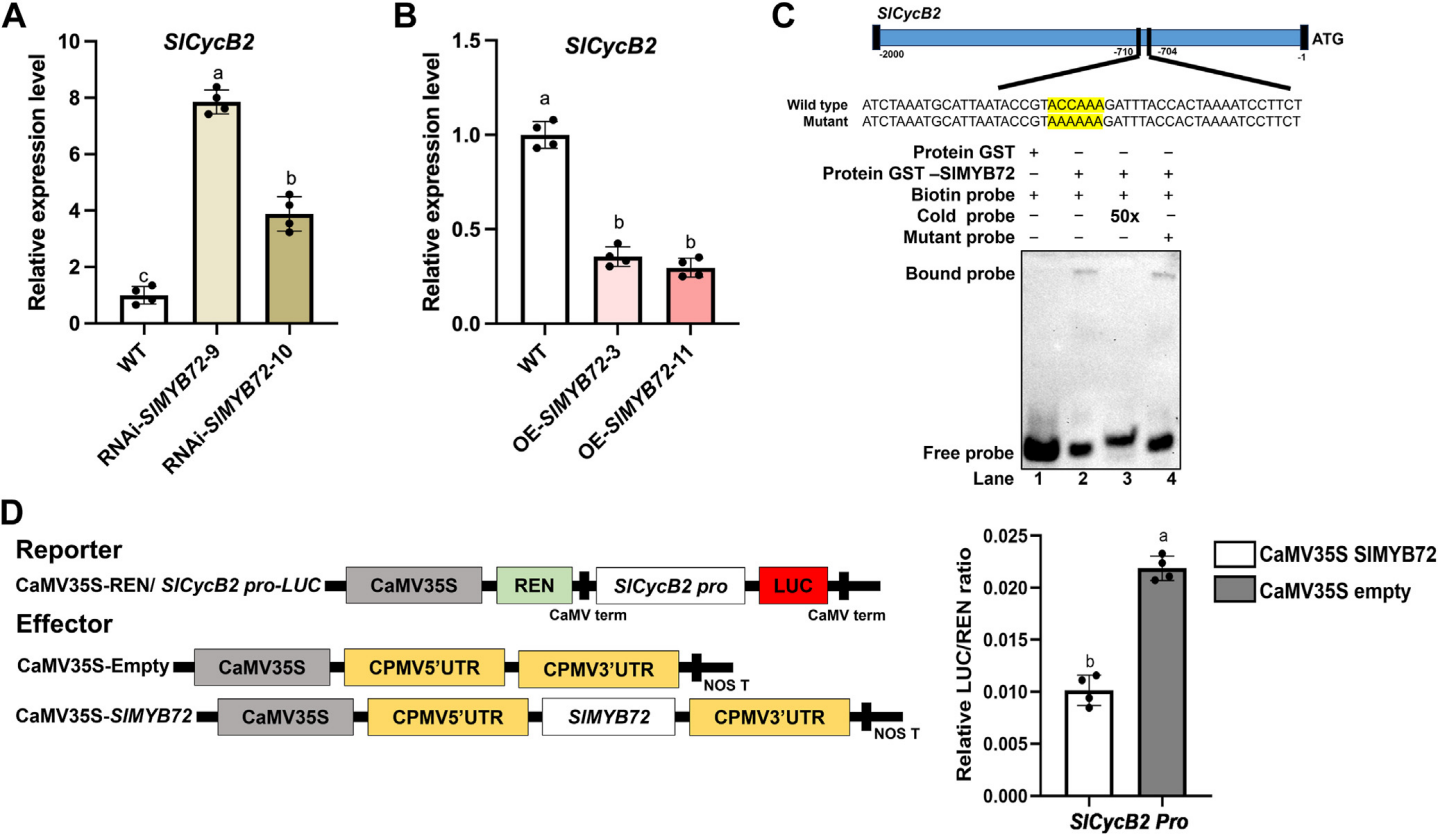
**SlMYB72 negatively regulates the expression of *SlCycB2*.** RT-qPCR analysis of *SlCycB2* expression in (*A*) RNAi-*SlMYB72* and *B*, OE-*SlMYB72* plants. Data are presented as the mean ± SD of at least three biological replicates. *C*, the EMSA assay indicated the binding of SlMYB72 to the cis-element of the *SlCycB2* promoter (*SlCycB2* pro). The unlabeled fragment served as a competitor (cold probe). The biotin-labeled DNA probe and mutant probe are about 0.02 μM, and the cold probe is about 1 μM (50 times of biotin probe). For each lane, the amount of proteins were equal and about 2 μg. – and + represent “absence” and “presence”, respectively. The MYB-binding motif in the *SlCycB2* promoter is indicated with a yellow background. *D*, diagrammatic representation of reporter and effector constructs of the dual-luciferase reporter assay (*left*). The influence of SlMYB72 on *LUC* expression driven by the *SlCycB2* promoter was determined using a dual-luciferase assay (*right*). Data are presented as mean ± SD of at least three biological replicates.

*SlCycB2* encodes a putative B-type cyclin that functions as a repressor of tomato trichome initiation, and its overexpression results in a notable reduction in all non-glandular, and type I/VI glandular trichomes (28). Given that SlMYB72 promotes the formation of type II, V, and VI trichomes, we further explored whether SlMYB72 regulates trichome formation by affecting the expression of *SlCycB2*. RT-qPCR analysis showed that the expression of *SlCycB2* was significantly elevated in RNAi-*SlMYB72* lines (Fig. 2*A*) and decreased in OE-*SlMYB72* lines compared to WT (Fig. 2*B*), suggesting that SlMYB72 negatively regulates the expression of *SlCycB2*. Sequence analysis of the *SlCycB2* promoter indicated the presence of an MYB-binding motif (ACCAAA) (24, 29, 30), and electrophoretic mobility shift assay (EMSA) was performed to confirm the binding of SlMYB72 with *SlCycB2* promoter. EMSA results demonstrated that co-incubation of the GST-SlMYB72 fusion protein and biotin-labeled probe containing the ACCAAA element of the *SlCycB2* promoter fragment produced a mobility shift. This mobility shift was eliminated when an unlabeled *SlCycB2* promoter fragment (cold probe) was supplemented as a competitor. However, this shift persisted when an unlabeled mutant probe (the ACCAAA motif of the *SlCycB2* promoter was mutated to AAAAAA) was added (Fig. 2*C*). Meanwhile, no mobility shift was observed in the case of co-incubation with GST protein and the biotin-labeled probe (Fig. 2*C*). These results indicate that SlMYB72 could bind to the *SlCycB2* promoter.

A dual-luciferase reporter assay was performed to evaluate the effect of SlMYB72 on the activity of the *SlCycB2* promoter. The coding region of *SlMYB72* was cloned into an effector vector and driven by CaMV35S. *SlCycB2* promoters were ligated to reporter constructs to control the expression of *luciferase* (*LUC*) gene (Fig. 2*D*). Effector and reporter constructs were co-expressed in *Nicotiana benthamiana* leaves and used to quantify LUC activity. The result showed that SlMYB72 significantly inhibited LUC activity compared to the empty vector, indicating that SlMYB72 represses the expression of *SlCycB2* (Fig. 2*D*). These results suggest that SlMYB72 targets the *SlCycB2* promoter and inhibits its transcription.

### Interference of *SlbHLH150* or *SlTTG1* has minimal impact on trichome formation

In Arabidopsis, several R2R3-MYB transcription factors have been proposed to form MBW complexes with bHLHs (GL3/EGL3/TT8) and TTG1 (a WD40 repeat protein) to regulate trichome initiation and flavonoid biosynthesis (10, 31). It is possible that SlMYB72 (R2R3-type) also forms MBW complex and further participates in tomato trichome formation. To address potential bHLHs and WD40 proteins that could form the MBW complex with SlMYB72, proteins homologous to Arabidopsis bHLH (GL3/TT8) and TTG1 in tomatoes were investigated in this study. As reported previously, the homologous proteins of *Arabidopsis* GL3, TT8 and TTG1 are SlGL3 (Solyc08g081140), SlbHLH150 (Solyc09g065100, SlTT8) and SlTTG1 (Solyc03g097340), respectively (32). Since overexpression of *SlGL3* plays a limited role in the number and branching of *Arabidopsis* trichomes, it seems that SlGL3 might have less function in trichome formation in tomatoes (33). Therefore, SlbHLH150 (Solyc09g065100) and SlTTG1 (Solyc03g097340) were selected for further studies.

**Figure 3:**
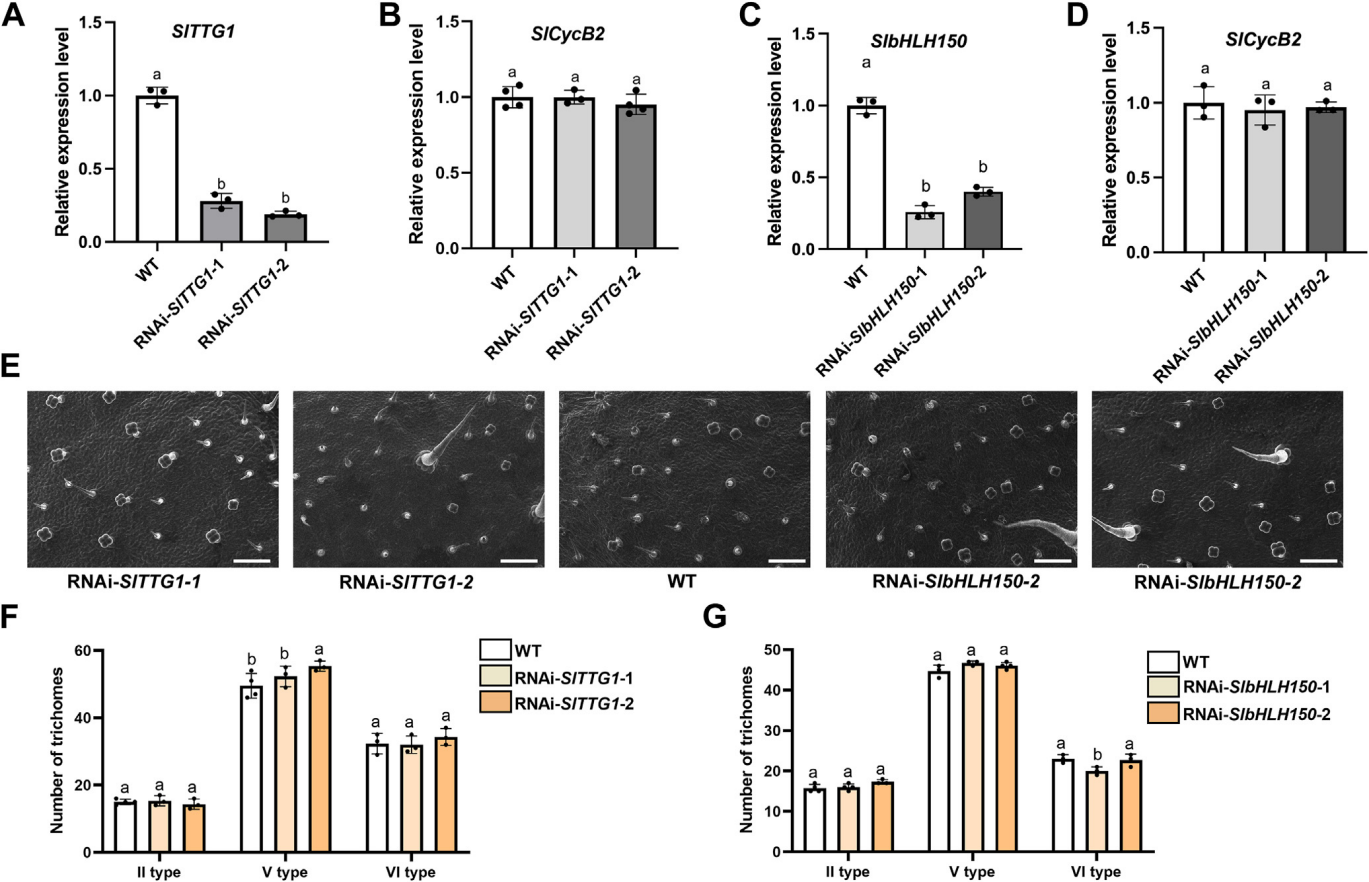
**Interference of *SlbHLH150* or *SlTTG1* did not affect trichome formation.***A*, RT-qPCR analysis of *SlTTG1* and (*B*) *SlCycB2* expression in the RNAi-*SlTTG1* plants. *C*, RT-qPCR analysis of *SlbHLH150* and (*D*) *SlCycB2* expression in RNAi-*SlbHLH150* plants. Data are presented as mean ± SD of at least three biological replicates. *E*, scanning electron micrographs of trichomes on the surface of tomato leaves of RNAi-*SlTTG1* and RNAi-*SlbHLH150* plants. Scale bars = 50 mm. *F*, trichome counting in the RNAi-*SlTTG1* and (*G*) RNAi-*SlbHLH150* lines. The number of type II trichomes within a 0.5 cm^2^ region and types V and VI trichomes within a 2.2 mm^2^ region of the leaves were counted. Data were obtained from at least three biological replicates. Distinct lowercase letters indicate statistically significant differences (*p* < 0.05).

To investigate the potential role of SlbHLH150 and SlTTG1 in trichome formation, *SlbHLH150* and *SlTTG1* interference lines were generated. For subsequent analysis of trichome formation, independent RNAi plants exhibiting significantly reduced expression of *SlTTG1* (Fig. 3*A*) and *SlbHLH150* (Fig. 3*C*) were selected for further analysis of trichome formation. RT-qPCR analysis revealed that interference with *SlTTG1* or *SlbHLH150* did not affect the expression of *SlCycB2* (Fig. 3, *B* and *D*). The density of trichomes on the leaves of RNAi-*SlTTG1* and RNAi-*SlbHLH150* plants was indistinguishable from that of WT tomato leaves based on the results of scanning electron microscopy (Fig. 3*E*). Moreover, the number of type II, V, and VI trichomes on the leaves of the WT, RNAi-*SlTTG1*, and RNAi-*SlbHLH150* lines were counted, and the results showed no significant differences among these plants (Fig. 3, *F* and *G*). These results indicate that interference of *SlbHLH150* or *SlTTG1* has minimal effect on trichome formation.

**Figure S2:**
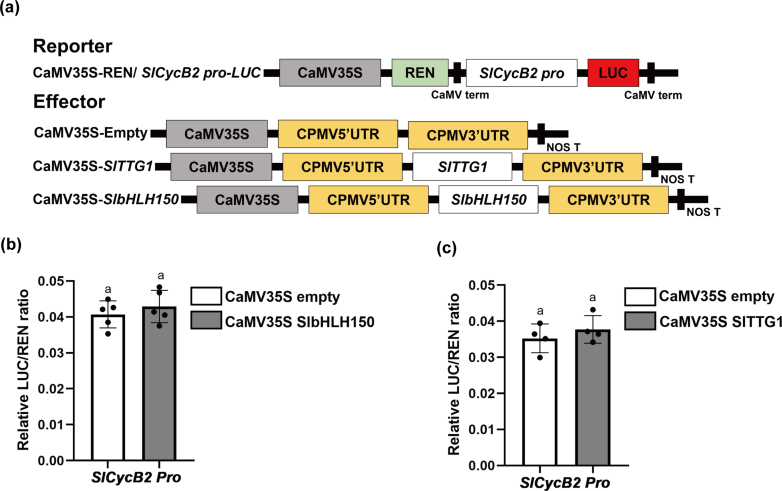
**Figure S2. Transcriptional control of SlTTG1 and SlbHLH150 on the activity of the *SlCycB2* promoter using a dual-luciferase reporter assay.** (a) Diagrammatic illustration of the reporter and effector vectors for the dual-luciferase reporter assay. (b) Effect of SlbHLH150 and (c) SlTTG1 on the *SlCycB2* promoter activity. Data are presented as mean ± SD of at least three biological replicates.

To verify the function of SlbHLH150 and SlTTG1 in trichome formation, we analyzed the effect of these proteins on *SlCycB2* expression using dual-luciferase reporter assays. The *LUC* gene driven by *SlCycB2* promoter was co-expressed with *SlbHLH150* or *SlTTG1* genes controlled by the CaMV35S promoter in *N. benthamiana* leaves to quantify LUC activity (Fig. S2). The result demonstrated no significant changes in *SlCycB2* promoter-driven *LUC* expression regardless of the presence of SlbHLH150 and SlTTG1 (Fig. S2). These results suggest that SlbHLH150 and SlTTG1 alone do not affect the expression of *SlCycB2*. It is probable that SlbHLH150 and SlTTG1 are not predominant factors in trichome formation and they might form MBW complex with R2R3-type MYBs to perform regulatory functions.

### SlMYB72 interacts with SlbHLH150 and SlTTG1 to form MBW complex

**Figure 4:**
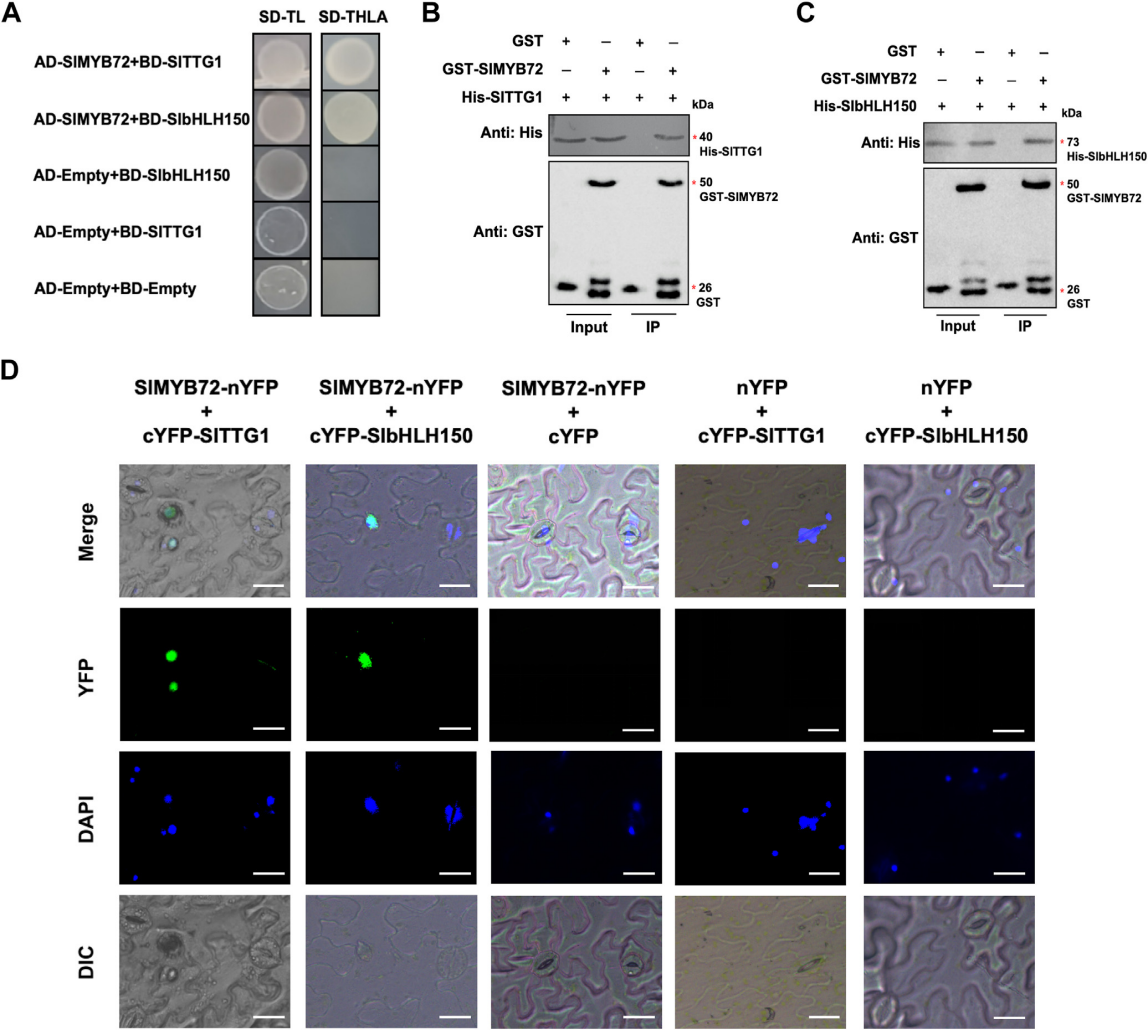
**SlMYB72 interacts with SlbHLH150 and SlTTG1.** Verification of the interaction between SlMYB72, SlTTG1 and SlbHLH150 using (*A*) Y2H, (*B* and *C*) GST pull-down, and (*D*) BiFC assays. In the Y2H assay, the groups of AD-empty and BD-empty, AD-empty and BD-SlbHLH150, and AD-empty and BD-SlTTG1 were used as negative controls. SD-TL is a selective dropout medium without Trp and Leu. SD-THLA is a selective dropout medium without Trp, Leu, His, and Ade. In GST pull-down assays, GST, GST-fusion proteins, and His-fusion proteins were distinguished by immunoblotting using antibodies against GST and His tags, respectively. Equal amounts of GST and GST-SlMYB72 were loaded in each lane for GST antibodies. The loading amount of the IP sample is five times that of the input samples, which is further used to evaluate the amount of His-SlTTG1 or SlbHLH150 pulled down by GST-SlMYB72 using the antibody against his tag. In the BiFC assay, the nuclei were stained with DAPI, and YFP fluorescent signals were determined by confocal microscopy. Scale bars = 20 mm.

The MBW complex plays a crucial regulatory role in *Arabidopsis* trichome initiation (19). To examine whether SlbHLH150 and SlTTG1 could form an MBW complex with SlMYB72, the interactions between SlbHLH150, SlTTG1, and SlMYB72 were validated. The results of the yeast-two-hybrid (Y2H) assay showed that yeast cells co-transformed with AD-SlMYB72 and BD-SlbHLH150 or BD-SlTTG1 were able to grow normally on selective dropout medium without Trp, His, Leu, and Ade (SD-THLA) (Fig. 4*A*), demonstrating that SlMYB72 interacts with both SlbHLH150 and SlTTG1. Subsequently, GST (glutathione-S-transferase) pull-down assays were performed to confirm these interactions. The results indicated that GST-SlMYB72 fusion proteins, instead of GST itself, could capture His-SlTTG1 and His-SlbHLH150, respectively (Fig. 4, *B* and *C*). Results from biomolecular fluorescence complementation (BiFC) assays revealed that *N. benthamiana* leaves co-expressing N-terminal YFP-labeled SlMYB72 (SlMYB72-nYFP) and C-terminal YFP-labeled SlTTG1 (cYFP-SlTTG1) or cYFP-SlbHLH150 emitted YFP fluorescent signals that overlapped with 4′,6-diamidino-2-phenylindole (DAPI) staining (Fig. 4*D*). These results emphasize the interaction of SlMYB72 with SlTTG1 and SlbHLH150.

**Figure 5:**
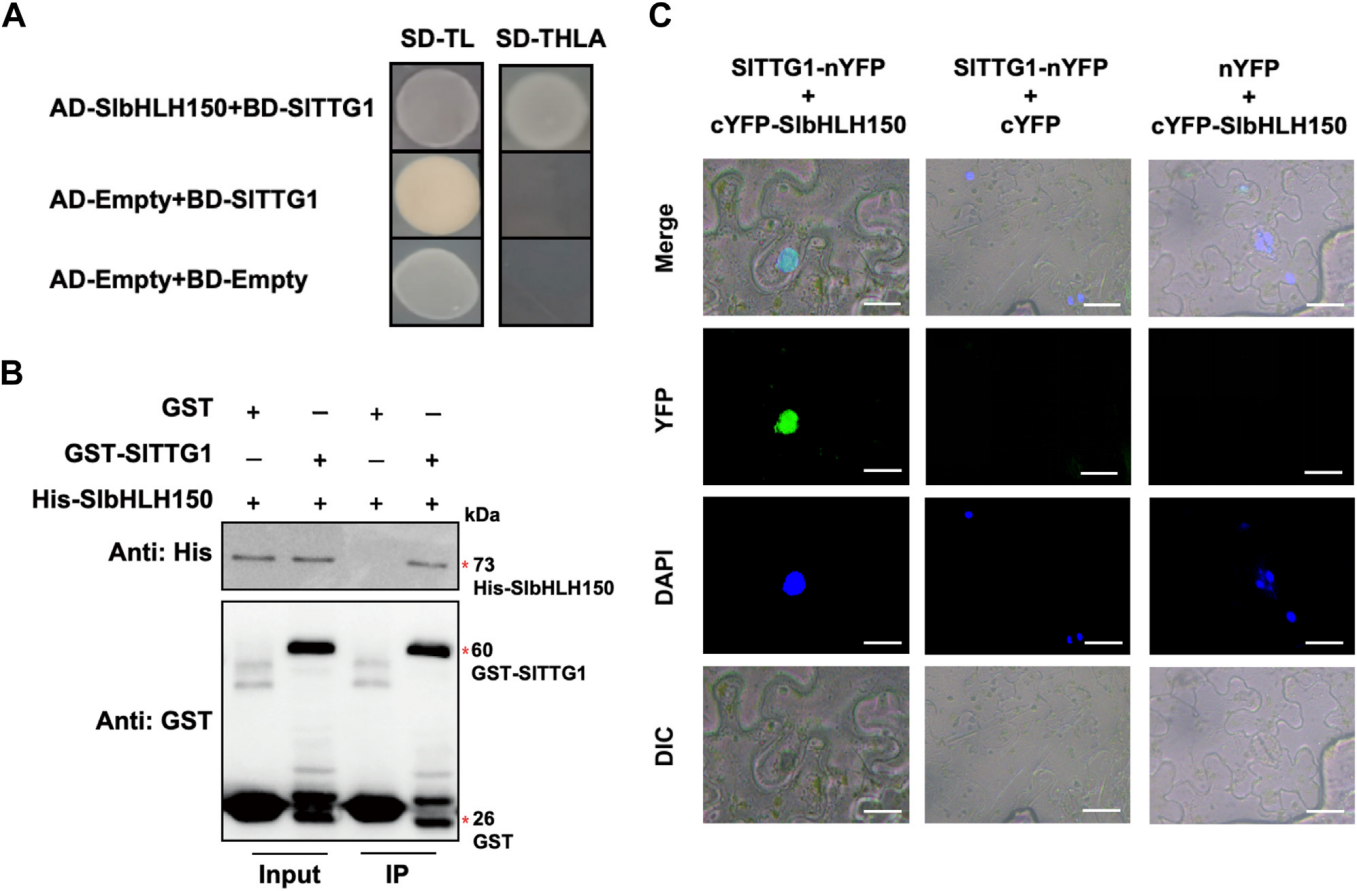
**SlbHLH150 interacts with SlTTG1.** Identification of the interaction between SlTTG1 and SlbHLH150 using (*A*) Y2H, (*B*) GST pull-down, and (*C*) BiFC assays. The groups of AD-empty and BD-empty, and AD-empty and BD-SlTTG1 were used as negative controls in the Y2H assay. Antibodies against His and GST tags were used for immunoblotting to analyze the His-fusion proteins and GST-fusion proteins (or GST itself) in GST pull-down assays, respectively. Equal amounts of GST and GST-SlTTG1 were loaded in each lane for GST antibodies. The loading amount of the IP sample is five times that of input samples, which is further used to evaluate the amount of SlbHLH150 pulled down by GST-SlTTG1 using the antibody against his tag. In the BiFC assay, the nuclei were indicated by DAPI staining and YFP fluorescent signals were observed by confocal microscopy. Scale bars = 20 mm.

We further determined the interaction of SlbHLH150 with SlTTG1 using Y2H, GST pull-down, and BiFC assays. The result from the Y2H assay showed that yeast cells co-transformed with AD-SlbHLH150 and BD-SlTTG1 could survive on SD-THLA medium (Fig. 5*A*). His-SlbHLH150 could be precipitated by GST-SlTTG1 rather than by GST alone (Fig. 5*B*). Additionally, YFP fluorescent signals were observed in *N. benthamiana* leaves transiently co-transfected with SlTTG1-nYFP and cYFP-SlbHLH150 (Fig. 5*C*). These results demonstrate the interaction of SlTTG1 with SlbHLH150. Overall, these findings indicate that SlMYB72, SlTTG1, and SlbHLH150 can interact with each other and they tend to form an MBW ternary complex through pairwise interactions.

### SlMYB75 interacts with SlbHLH150 and SlTTG1 to form MBW complex

**Figure 6:**
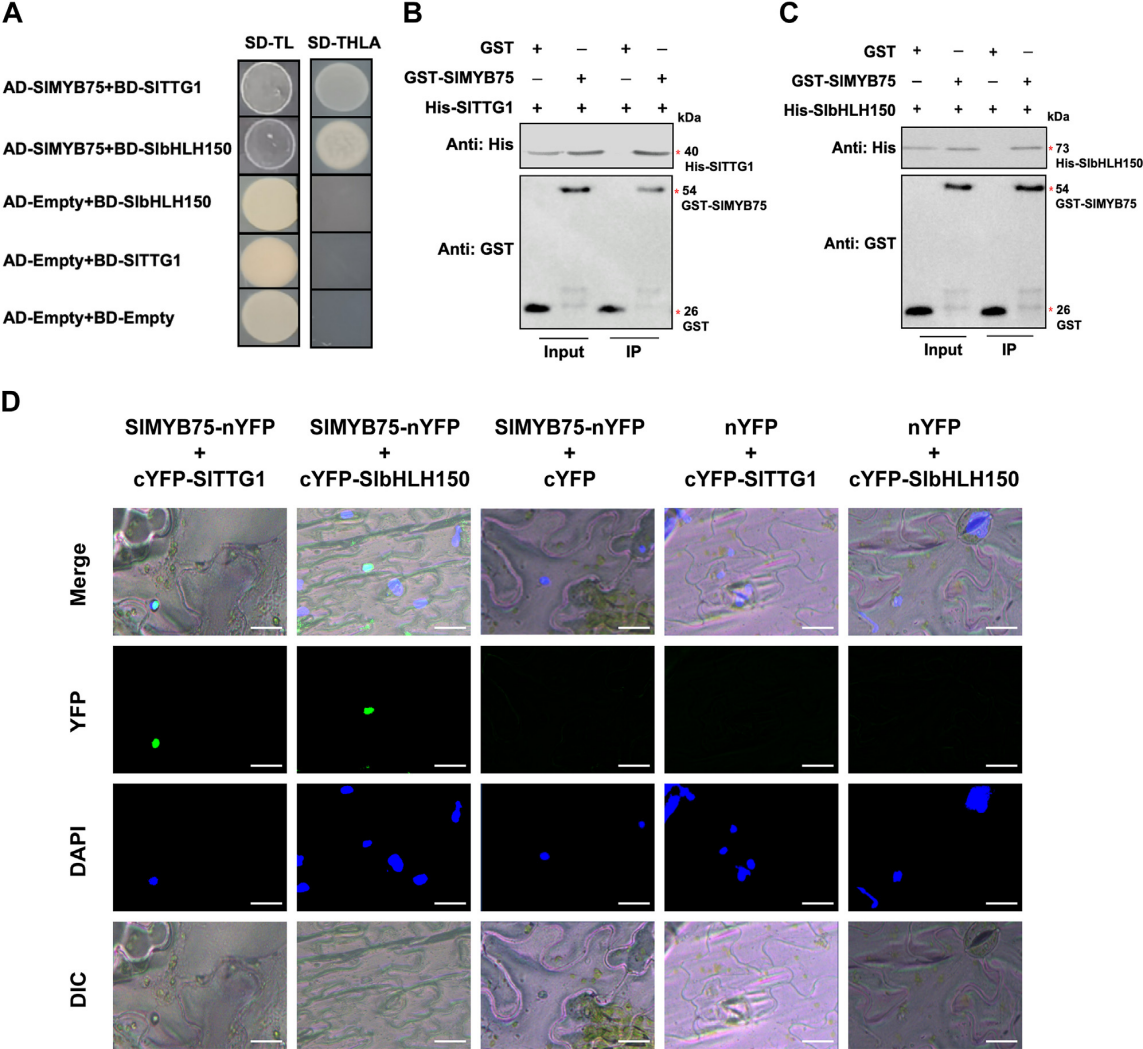
**SlMYB75 interacts with SlbHLH150 and SlTTG1.** Determination of the interaction between SlMYB75, SlTTG1, and SlbHLH150 using (*A*) Y2H, (*B* and *C*) GST pull-down, and (*D*) BiFC assays. The groups of AD-empty and BD-SlbHLH150, AD-empty and BD-SlTTG1, and AD-empty and BD-empty were used as negative controls in the Y2H assay. His-fusion proteins, GST, and GST-fusion proteins were recognized by immunoblotting using antibodies against His and GST tags in GST pull-down assays, respectively. Equal amounts of GST and GST- SlMYB75 were loaded in each lane for GST antibodies. The loading amount of the IP sample is five times that of input samples, which is further used to evaluate the amount of His-SlTTG1 or SlbHLH150 pulled down by GST-SlMYB75 using the antibody against his tag. YFP fluorescent signals and the DAPI-stained nuclei were determined by confocal microscopy in the BiFC assays. Scale bars = 20 mm.

Our previous study identified that SlMYB75, an R2R3-type MYB transcription factor, promotes the *SlCycB2* expression and negatively regulates the trichome formation in tomato (34). To determine whether SlMYB75 could also form an MBW complex similar to SlMYB72, the interactions of SlMYB75 with SlTTG1 and SlbHLH150 were verified using Y2H, BiFC, and GST pull-down assays. Studies from the Y2H assay described that the introduction of AD-SlMYB75 and BD-SlTTG1 or BD-SlbHLH150 into yeast cells ensured their growth on the SD-THLA medium (Fig. 6*A*). The results of GST pull-down assays indicated that His-SlTTG1 and His-SlbHLH150 were pulled down by GST-SlMYB75 rather than by GST alone (Fig. 6, *B* and *C*). BiFC assays demonstrated that YFP fluorescent signals were detectable in *N. benthamiana* leaves injected with SlMYB75-nYFP and cYFP-SlTTG1 or cYFP-SlbHLH150 (Fig. 6*D*). These results suggest that SlMYB75 interacts with both SlbHLH150 and SlTTG1. It is logical to assume that these three proteins form an MBW complex that regulates trichome formation.

**Figure S3:**
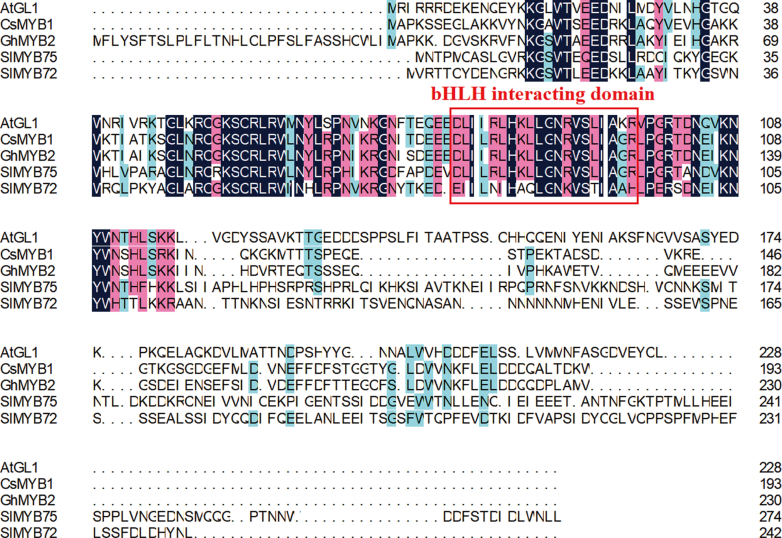
**Figure S3. Multiple sequence alignments of SlMYB72 and SlMYB75 with R2R3-type MYBs involved in trichome initiation.** AtGL1, CsMYB1 and GhMYB2 are GL1 proteins from *Arabidopsis thaliana*, *Camellia sinensis* and *Gossypium hirsutum*, respectively. The red box indicated specific residues ([D/E]Lx2[R/K]x3Lx6Lx3R) of MYBs contributing to interact with bHLHs, and these specific residues were marked as bHLH interacting domain.

It is noteworthy that R2R3 MYBs within the MBW complex generally possess the conserved motif “[D/E]Lx2[R/K]x3Lx6Lx3R”, which is responsible for interacting with bHLH transcription factors (35, 36). GL1 proteins are typical R2R3-type MYBs that can interact with bHLH and TTG1 to form MBW complex to regulate trichome formation. To ascertain whether SlMYB72 and SlMYB75 contain the conserved bHLH interacting motif, amino acid sequence alignments were performed between SlMYB72, SlMYB75, and GL1 proteins from *Arabidopsis* (AtGL1), *Camellia sinensis* (CsMYB1), and *Gossypium hirsutum* (GhMYB2). The results from multiple sequence alignments revealed that the bHLH-interacting domain ([D/E]Lx2[R/K]sx3Lx6Lx3R) is present in AtGL1, CsMYB1, GhMYB2, and SlMYB75, but absent in SlMYB72 (Fig. S3). Although SlMYB72 lacks this conserved domain, results from our biochemical experiments showed the interplay of SlMYB72 with SlbHLH150, suggesting that the interaction between SlMYB72 and SlbHLH150 does not rely on the conserved bHLH-interacting motif, as is the case for SlMYB75. Therefore, it is hypothesized that the interaction of SlMYB75 with SlbHLH150 might be stronger than that between SlMYB72 and SlbHLH150, owing to the presence of the conserved motif in SlMYB75 protein. Furthermore, there might be a competitive relationship between SlMYB72 and SlMYB75.

### SlMYB75 competes SlbHLH150 and SlTTG1 with SlMYB72

**Figure 7:**
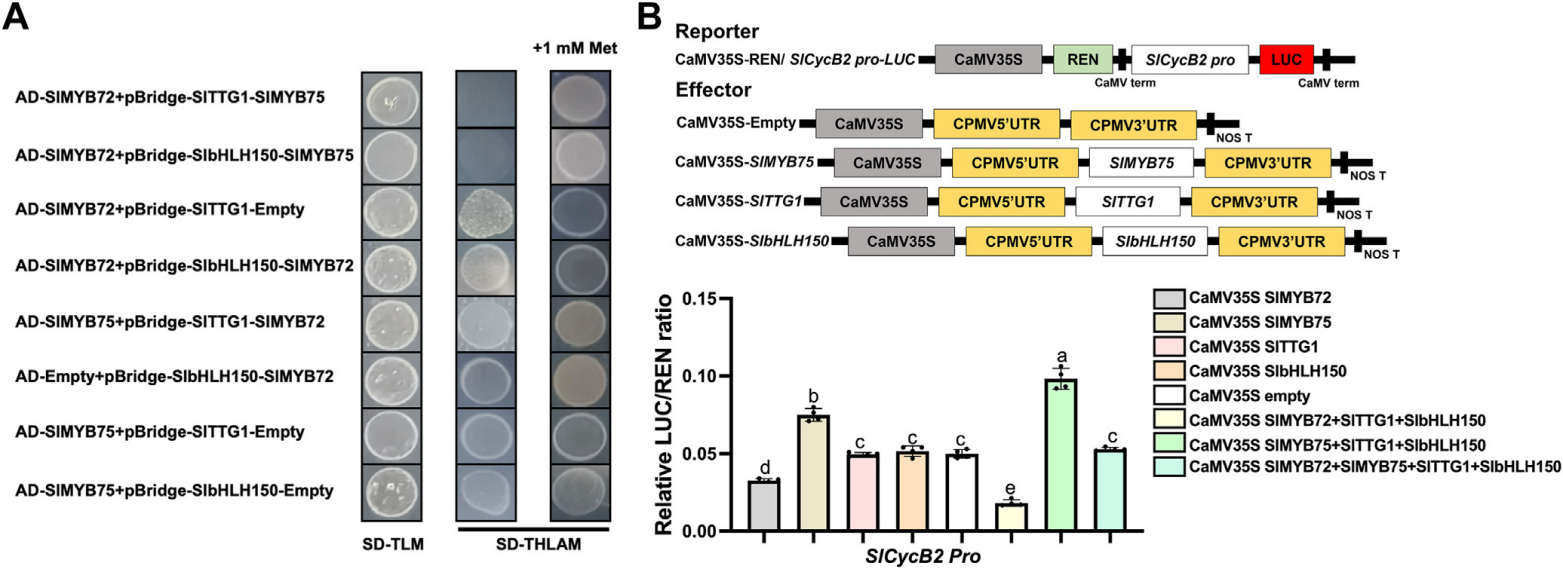
**SlMYB72 and SlMYB75 antagonistically regulate the expression of *SlCycB2*.***A*, assessment of the competitive interaction between SlMYB72 and SlMYB75 with SlbHLH150 and SlTTG1 using the Y3H assay. Expression of the bridge proteins SlMYB72 and SlMYB75 was repressed in the presence of methionine (Met) and induced in the absence of Met. SD-TLM is a selective dropout medium without Trp, Leu, and Met. SD-THLAM is a selective dropout medium without Trp, Leu, His, Ade, and Met. *B*, schematic representation of the reporter and effector vectors for dual-luciferase reporter assay and luciferase activity under the control of the *SlCycB2* promoter in the presence of SlMYB72, SlMYB75, SlbHLH150, and SlTTG1. Data are presented as mean ± SD of at least three biological replicates.

Although both SlMYB72 and SlMYB75 regulate trichome formation, they exert opposite effects on this process. Considering their contrasting regulatory functions in trichome formation and their respective binding properties with interactors, it is conceivable that there is competition between SlMYB75 and SlMYB72 for binding to SlbHLH150 and SlTTG1. To test this hypothesis, we performed a yeast-three-hybrid (Y3H) assay to validate the competitive relationship between SlMYB72 and SlMYB75. First, the coding regions of *SlMYB75* and *SlTTG1* (or *SlbHLH150*) were cloned into pBridge vector to express SlMYB75 (as a bridge protein) and BD-SlTTG1 (or BD-SlbHLH150) proteins. This pBridge construct was then co-transformed with AD-SlMYB72 into yeast cells, and the resulting cells successfully grew on SD-THLA medium supplemented with methionine (Met). However, these yeast cells were unable to survive on SD-THLA medium without Met (Fig. 7*A*), indicating that the expressed bridge protein SlMYB75, in the absence of Met, could effectively inhibit the interaction of AD-SlMYB72 with BD-SlTTG1, as well as BD-SlbHLH150. Conversely, the open reading frames of *SlMYB72* and *SlTTG1* (or *SlbHLH150*) were also ligated into pBridge vector to express SlMYB72 as a bridge protein and BD-SlTTG1 (or BD-SlbHLH150) proteins. The resulting pBridge construct was co-transformed with AD-SlMYB75 into yeast cells, and the resulting cells exhibited regular growth on SD-THLA medium with and without Met (Fig. 7*A*), implying that the expression of bridge protein SlMYB72 has no significant effect on the interaction of AD-SlMYB75 with BD-SlTTG1, as well as BD-SlbHLH150. These results reveal that SlMYB75 exhibits a more robust interaction with SlbHLH150 and SlTTG1 compared to SlMYB72. Therefore, SlMYB75 competes with SlMYB72 for binding to SlbHLH150 and SlTTG1.

### SlMYB72 and SlMYB75 antagonistically regulate trichome formation

It has been proven that the formation of MBW ternary complexes consisting of bHLH, WD40, and MYBs contributes to the activation of downstream genes regulated by MYBs (1). It is logical to hypothesize that SlMYB72 and SlMYB75 might enhance the regulation of *SlCycB2* by forming MBW complexes with SlbHLH150 and SlTTG1, and they function antagonistically in trichome formation. A dual-luciferase reporter assay was performed to evaluate the effect of the MBW complex composed of SlbHLH150, SlTTG1, and SlMYB72 (or SlMYB75) on the expression of *SlCycB2*. As illustrated in Figure 7*B*, the *LUC* gene was induced by the *SlCycB2* promoter. The expressions of *SlMYB72*, *SlMYB75*, *SlbHLH150*, and *SlTTG1* were regulated by the CaMV35S promoter. The findings from the dual-luciferase reporter assay indicated that overexpression of SlTTG1 or SlbHLH150 alone in *N. benthamiana* leaves had a negligible influence on LUC activity compared with the empty vector (Fig. 7*B*). Consistent with previous conclusions, LUC activity was repressed by SlMYB72 overexpression in *N. benthamiana* leaves, and this inhibitory effect was further enhanced in the presence of SlTTG1 and SlbHLH150 (Fig. 7*B*). Similarly, SlMYB75 overexpression stimulated LUC activity in *N. benthamiana* leaves, which was amplified in the presence of SlTTG1 and SlbHLH150 (Fig. 7*B*). In addition, co-expression of SlMYB72, SlMYB75, SlTTG1, and SlbHLH150 in *N. benthamiana* leaves exhibited identical LUC activity to that of the empty vector (Fig. 7*B*). These results indicate that the MBW complexes of SlMYB72 and SlMYB75 strengthen their regulatory role in *SlCycB2* expression. Furthermore, the observed opposite effects suggest that SlMYB72 and SlMYB75 antagonistically regulate trichome formation.

**Figure 8:**
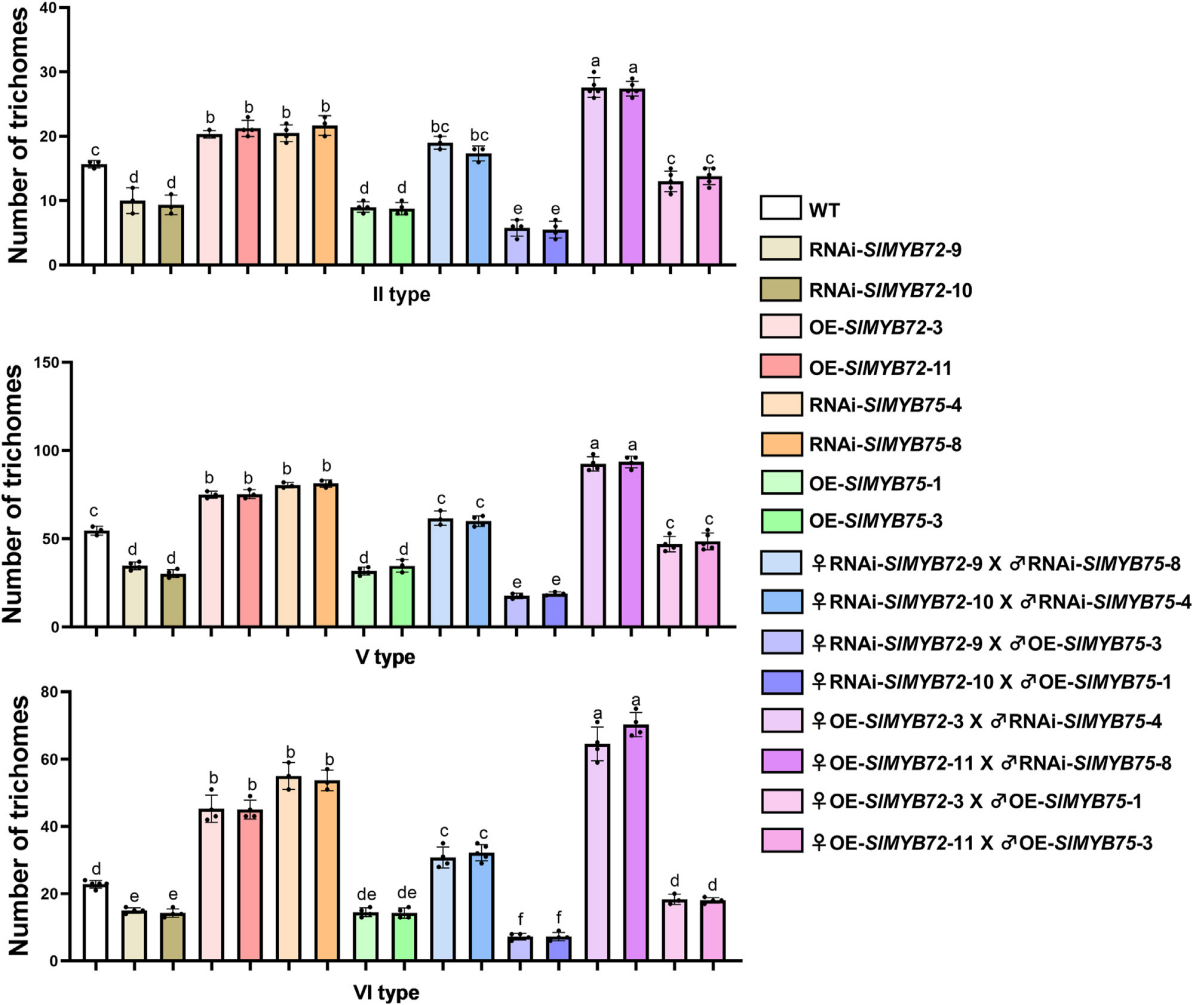
**Trichome counting in the T1 generation of genetic hybridization experiments.** The number of trichomes on the leaves of the hybrid progeny resulting from the genetic cross between *SlMYB72* and *SlMYB75* transgenic plants, and the corresponding parent plants of these progeny were determined. Specifically, the number of type II trichomes within a 0.5 cm^2^ area and the number of type V and type VI trichomes within a 2.2 mm^2^ area of the leaves were counted. Counting was performed with at least three biological replicates. Data were presented as the mean ± SD. Distinct lowercase letters represent statistically significant differences (*p* < 0.05).

**Figure S4:**
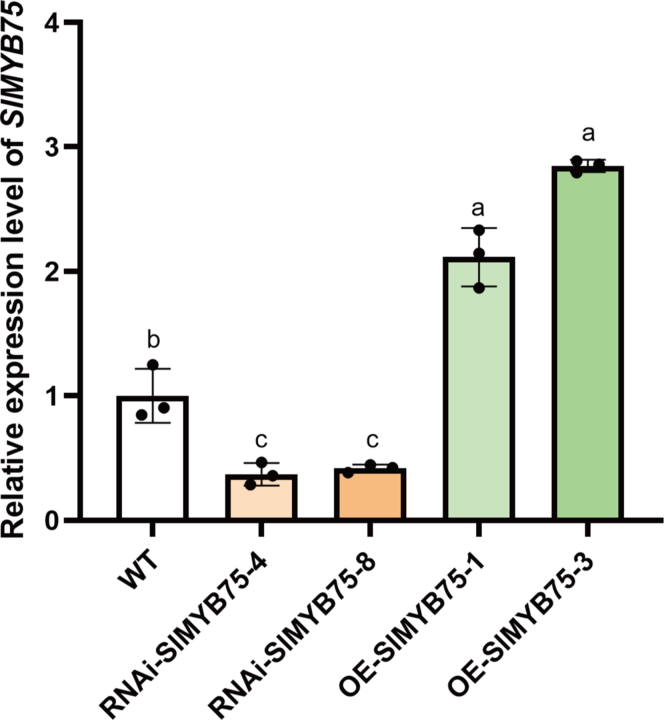
**Figure S4. RT-qPCR analysis of *SlMYB75* expression in RNAi-*SlMYB75* and OE- *SlMYB75* plants.** Data are presented as mean ± SD of at least three biological replicates.

**Figure S5:**
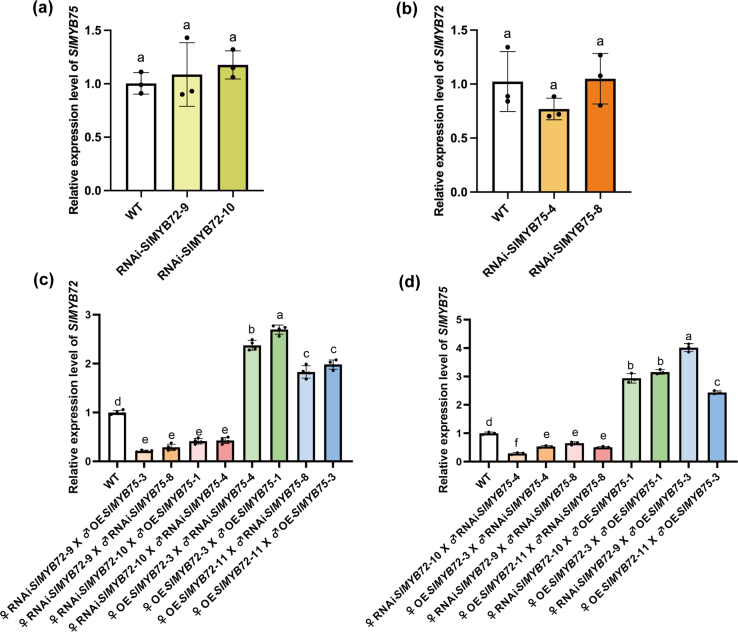
**Figure S5. Verification of *SlMYB72* and *SlMYB75* expression in hybrid plants using RT-qPCR.** (a) Relative expression of *SlMYB75* in *SlMYB72* transgenic plants. (b) Relative expression of *SlMYB72* in *SlMYB75* transgenic plants. (c) Relative expression of *SlMYB72 and* (d) *SlMYB75* in hybrid plants plants. Hybrid plants are generated by genetic crossing of *SlMYB72* and *SlMYB75* transgenic plants. Data are presented as mean ± SD of at least three biological replicates.

To further corroborate the antagonistic regulation of SlMYB72 and SlMYB75 in trichome formation, independent transgenic plants of *SlMYB72* (RNAi-*SlMYB72* and OE-*SlMYB72*) and *SlMYB75* (RNAi-*SlMYB75* and OE-*SlMYB75*) were selected for the genetic crossing experiments. The overexpression and interference of *SlMYB75* in RNAi-*SlMYB75* and OE-*SlMYB75* plants were controlled by 35S promoter and confirmed by qRT-PCR (Fig. S4). We also determined the expression of *SlMYB75* in RNAi-*SlMYB72* transgenic plants, and *SlMYB72* in RNAi-*SlMYB75* lines. The results showed that the expression of *SlMYB75* in RNAi-*SlMYB72* and *SlMYB72* in RNAi-*SlMYB75* were comparable to those in the wild type (Fig. S5, *A* and *B*). The T1 generations of hybrid plants with low *SlMYB72* and *SlMYB75* expression (♀RNAi-*SlMYB72*×♂RNAi-*SlMYB75*), high *SlMYB72* and *SlMYB75* expression (♀OE-*SlMYB72*×♂OE-*SlMYB75*), low *SlMYB72* and high *SlMYB75* expression (♀RNAi-*SlMYB72*×♂OE-*SlMYB75*), and high *SlMYB72* and low *SlMYB75* expression (♀OE-*SlMYB72*×♂RNAi-*SlMYB75*) were selected for trichome abundance analysis (Fig. S5, *C* and *D*). The findings indicated that the number of type II, V, and VI trichomes on the leaves of ♀RNAi-*SlMYB72*×♂OE-*SlMYB75* were significantly lower than those of their parental plants (RNAi-*SlMYB72* and OE-*SlMYB75*) and WT (Fig. 8). Conversely, trichomes on the leaves of ♀OE-*SlMYB72*×♂RNAi-*SlMYB75* were significantly higher than those of their parents (OE-*SlMYB72* and RNAi-*SlMYB75*) and WT. Furthermore, the abundance of trichomes on the leaves of ♀RNAi-*SlMYB72*×♂RNAi-*SlMYB75* and ♀OE-*SlMYB72*×♂OE-*SlMYB75* were comparable to those of the WT, which were distinctly different from those of their respective parental plants (Fig. 8). These results provide additional genetic evidence for the insights into the antagonistic regulation of SlMYB72 and SlMYB75 in trichome formation.

## Discussion

### MBW complexes regulate the formation of multicellular trichomes in tomato

The R2R3-MYBs, bHLH transcription factors, and WD40 repeat proteins are key regulators of trichome development and are characterized by their phenotypes affecting trichome formation (37). Ternary MBW complexes, composed of R2R3-MYBs, bHLH (GL3/EGL3), and WD40 protein (TTG1), enhance the expression of *GL2*/*TTG2*, thereby regulating the initiation of unicellular trichomes in *Arabidopsis* (10). Notably, this MBW complex-mediated regulatory mechanism of trichome formation has also been proposed in other plants with unicellular trichomes, such as cotton and tea (38, 39). Recent evolutionary analyses indicate that the aforementioned MBW complexes controlling trichome formation are conserved and present in common ancestors of flowering plants (40). However, the involvement of MBW complex in multicellular trichome formation for plants with multicellular trichomes (such as the model organism tomato) is rarely reported, despite the well-established functions of several R2R3-MYBs (*e.g.* SlMIXTA1, SlMYB75, and SlMYB52), and bHLH transcription factors (SlMYC1 and SlbHLH95) during trichome formation in tomatoes (7). Our present study demonstrates the regulatory functions of MBW complexes, which are composed of R2R3-MYB (SlMYB72 or SlMYB75), bHLH (SlbHLH150), and WD40 repeat protein (SlTTG1), in the formation of tomato multicellular trichome. This MBW-regulated trichome formation in tomato is analogous to that in *Arabidopsis*, revealing the correlation and evolutionary consistency in the regulatory mechanisms between unicellular and multicellular trichomes and providing a theoretical basis for understanding trichome formation in other plants.

### SlMYB72 and SlMYB75 are key regulators of both flavonoid biosynthesis and trichome formation in tomato

In addition to its predominant function in trichome formation, the MBW complex plays an essential role in regulating vacuolar acidification and phenylpropanoid biosynthesis, including flavonoids and anthocyanins (22, 41, 42). Many studies have elucidated the regulatory role of the MBW complex in flavonoids and anthocyanin biosynthesis in various plants, such as *Arabidopsis*, persimmon, pear, and tomato (32, 43, 44, 45). A previous study on tomatoes revealed that R2R3-MYBs (SlANT1 and SlAN2), bHLHs (SlGL3 and SlTT8), and WD40 protein (SlAN11, also named SlTTG1 in this study) can form the MBW complex to regulate *SlDFR* expression, thereby adjusting anthocyanin accumulation in stems and leaves (32). These studies indicate the vital functions of SlTT8 (also named SlbHLH150 in this study) and SlTTG1 in phenylpropanoid biosynthesis. Moreover, previous works revealed the regulatory role of SlMYB72 and SlMYB75 in flavonoid metabolism (24, 46). In this study, SlMYB72 and SlMYB75 were further characterized for their roles in regulating trichome formation in tomatoes by forming MBW complex with SlTTG1 and SlbHLH150. Consequently, it is reasonable to propose that the MBW complexes (SlMYB72-SlbHLH150-SlTTG1 and SlMYB75-SlbHLH150-SlTTG1) identified in this study may also be involved in the regulation of phenylpropanoid biosynthesis, particularly flavonoid biosynthesis, which requires further exploration.

### SlMYB72 and SlMYB75 form MBW complexes that function as the activator and repressor of trichome formation in tomatoes, respectively

MYB family transcription factors are classified into four subfamilies based on the repeats of their DNA-binding domains, and they regulate numerous processes, including secondary metabolism and developmental responses (47, 48). Most of these MYBs serve as activators, while an increasing number of R2R3-type and R3-type MYBs have been recognized as key repressors of the phenylpropanoid pathway and trichome formation (2, 49). They usually compete with the R2R3-MYB activator of the MBW complex for binding to bHLHs, thereby inhibiting the expression of genes responsible for phenylpropanoid biosynthesis or trichome formation. For instance, the MBY repressors FhMYBx (R3-type) and FhMYB27 (R2R3-type) impede the assembly of the positive MBW complex by titrating bHLH proteins and substituting the activator R2R3-MYB FhPAP1 in the MBW complex, respectively, thus managing anthocyanin biosynthesis in *Freesia* flowers (50). It has also been extensively reported that *Arabidopsis* R3-type MYB repressors (CPC, TRY, ECT1, and TCL1) disrupt the MBW complex by competing with R2R3-MYBs for binding to EGL3/Gl3 and controlling trichome initiation (19). To date, there have been limited studies on R2R3-type MYB repressors acting on the MBW complex during trichome development. In addition, most MBW complexes have been reported to positively fine-tune trichome formation, whereas research on MBW complexes negatively mediating trichome formation has not been addressed (8). This gap is filled by our study, which not only proves the formation of positive (SlMYB72-SlbHLH150-SlTTG1) and negative (SlMYB75-SlbHLH150-SlTTG1) regulatory MBW complexes involved in trichome formation but also identifies their antagonistic regulatory relationship.

### The antagonistic regulation of SlMYB72 and SlMYB75 in tomato trichome formation may be mediated by auxin signaling

Although SlMYB72 and SlMYB75 antagonistically regulate trichome formation, it remains uncertain which factor plays a predominant regulatory role in this process and how they compete for SlbHLH150 and SlTTG1. Phytohormones are important regulators of trichome formation (51). Our previous studies demonstrated that auxin treatment induces the formation of type II, V, and VI trichome in tomato leaves, and the auxin-responsive factor SlARF4 plays a positive role in trichome formation by directly targeting R2R3-MYBs (SlTHM1 and SlMYB52), which can bind to the *SlCycB2* promoter and promote its expression (52). Furthermore, SlMYB72 interacts with SlARF4 and mediates the biosynthesis of chlorophyll, carotenoids, and flavonoids (24). SlARF4 can also target *SlMYB75* promoters and inhibit its gene expression, thus regulating trichome formation and sesquiterpene accumulation (34). These reports reveal that the functions of SlMYB72 and SlMYB75 are mediated by auxin and its responsive factor, SlARF4. Therefore, it is hypothesized that the action of MBW complexes identified in this study is probably regulated by auxin signaling to balance the formation of trichomes in tomatoes, and this hypothesis requires an in-depth explanation in the future.

It is worth noting that SlCycB2 is considered a repressor of trichome initiation based on the discovery from *SlCycB2* overexpression lines, in which almost all non-glandular trichomes, as well as the glandular ones, disappeared (28). However, the interference of *SlCycB2* resulted in an increase in type III and V trichomes, and the almost disappearance of type I trichomes (28). Intriguingly, a recent study showed that the knockout mutant of *SlCycB2* displayed an obviously increased number of digitate trichomes (types I-V), and a reduced number of peltate trichomes (types VI and VII) (53). These studies suggest that the exact role of SlCycB2 in trichome formation is contradictory, and requires further investigation in the future.

### The regulatory roles of SlbHLH150 and SlTTG1 in tomato trichome formation largely depend on the formation of MBW complex with SlMYB72 and SlMYB75

In addition to R2R3-MYBs, bHLH transcription factors (GL3/EGL3/TT8) and WD40 protein (TTG1), essential components of the MBW complex, also play a significant role in trichome development (5). In *Arabidopsis*, *ttg1* mutants lost trichomes, and the mutation of *GL3* caused fewer trichomes compared to WT (18, 54). The *egl3* mutant had no obvious trichome deficiency, but the *gl3 egl3* double mutant exhibited a glabrous phenotype (13). Furthermore, some small trichome primordium-like structures were observed on the leaf lamina of *ttg1* and *gl3 egl3* mutants under phytohormone treatment. However, this structure disappeared from the leaf lamina of *gl3 egl3 tt8* triplet mutant (16). These studies indicate the functional redundancy of these bHLH transcription factors (GL3, EGL3, and TT8). Notably, overexpression of tomato *GL3* (*SlGL3*) in *Arabidopsis* did not affect the number and branching of trichomes, implying that the function of SlGL3 may be distinct from that of *Arabidopsis* GL3 (33). In tomatoes, the roles of SlGL3/EGL3/TT8 and SlTTG1 in trichome formation have not yet been determined, although SlGL3/TT8 and SlTTG1 regulate anthocyanin accumulation in the form of the MBW complex (32). In contrast to *Arabidopsis*, the present study reveals that the interference of *SlTT8* (*SlbHLH150* in this study) or *SlTTG1* has no obvious effect on trichome density on the surface of tomato leaves. One possible explanation is that the residual low abundance of SlTT8 or SlTTG1 proteins in the corresponding interfering lines can ensure the formation of a functional MBW complex to maintain trichome formation. SlTT8 and SlTTG1 serve as enhancers of R2R3-MYBs in the MWB complex rather than functioning independently. Additionally, there might be several bHLHs and WD40 proteins with redundant functions of SlTT8 and SlTTG1 in tomatoes, therefore, the knockdown of *SlTTG8* or *SlTTG1* results in no significant deficiency in trichome formation. Nevertheless, it is important to explore the exact functions of SlTTG8 and SlTTG1 using relative knockout mutants created by the CRISPR method.

**Figure 9:**
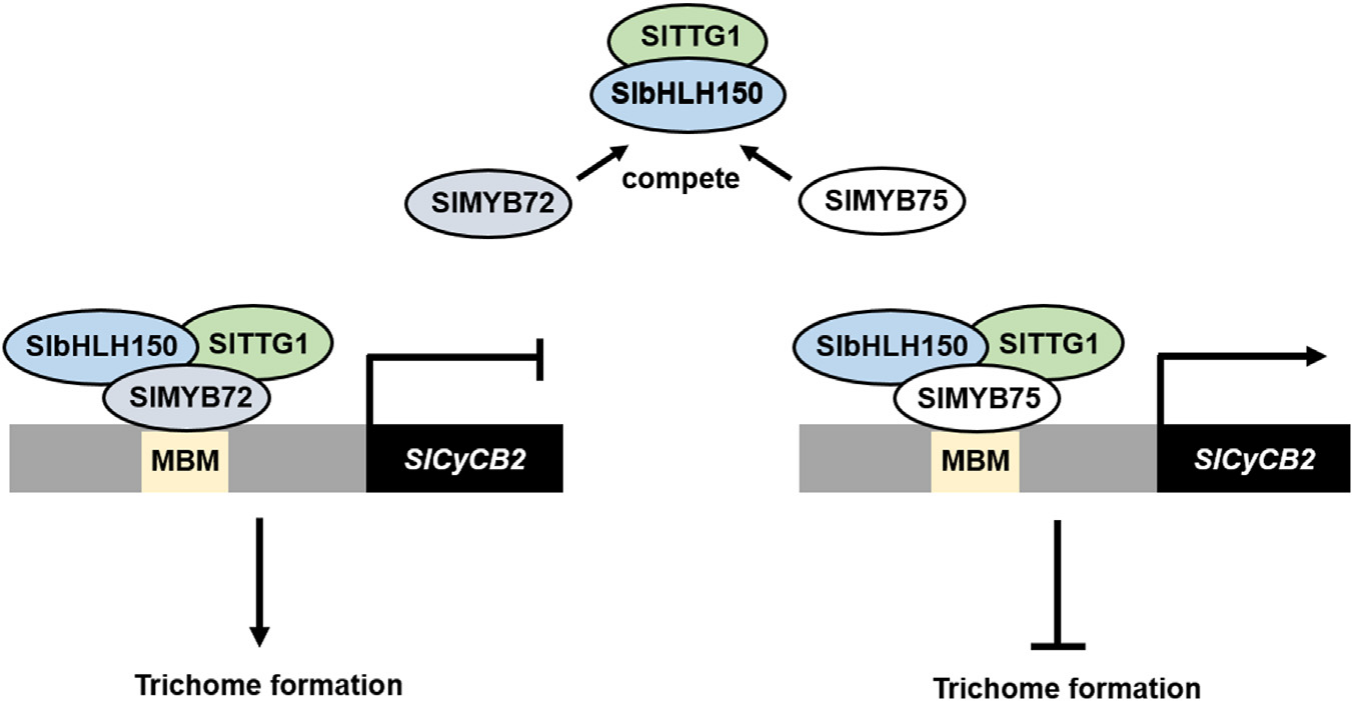
**Working model for the regulatory role of MBW complexes in tomato trichome formation.** First, SlMYB72 directly binds to the *SlCycB2* promoter and inhibits its expression, thus relieving the inhibitory effect of SlCycB2 to promote trichome formation in tomato leaves. SlMYB75 can target the promoter of *SlCycB2* and promote its expression, thereby enhancing the inhibitory effect of SlCycB2 to repress trichome formation in tomato leaves. Furthermore, SlbHLH150 and SlTTG1 can form MBW complexes with SlMYB72 or SlMYB75 to enhance their transcriptional activities, respectively. Moreover, SlMYB75 can compete with SlbHLH150 and SlTTG1 with SlMYB72 to balance the formation of trichome. MBM, MYB binding motif.

In conclusion, this study demonstrates that SlMYB72 and SlMYB75 could form the MWB complexes with SlbHLH150 and SlTTG1, and they antagonistically regulate the expression of *SlCycB2* to balance the formation of type II, V, and VI trichomes in tomato (Fig. 9). This improves our understanding of the regulatory mechanism of the MBW complex in multicellular trichome formation in tomatoes and other plant species and contributes to the development of pest-resistant plants by interfering with the trichome formation pathway using genetic engineering technology.

## Experimental procedures

### Plant material and growth conditions

Miniature tomato (*Solanum lycopersicum* ‘Micro-Tom’) was used in this study. Seeds from transgenic plants were sterilized with a 5% NaClO solution for 10 min and rinsed thrice with sterile water. Then, seeds were transferred to ½-strength Murashige & Skoog (½MS) growth medium containing kanamycin (100 mg/ml) for 10 days. Finally, the plants were transplanted into containers filled with nutritional soil and maintained in a growth chamber at 26 °C on a light and dark cycle of 14  and 10 h, respectively. The relative humidity was maintained at 70%, and the light intensity was set at 250 μmol photons/m^2^/s.

### Trichome counts and phenotyping

The number of trichomes on the surface of the tomato leaves was counted as described previously (34). Fully expanded leaves of wild-type (WT) and transgenic plants with similar pre-flowering status were collected for trichome counts. Strips measuring 6 mm × 15 mm were dissected from the same position on the collected leaves and examined for type II, V, and VI trichomes on the surface using a JNOEC JSZ5B stereomicroscope or Hitachi TM400plus scanning electron microscope. One strip per leaf was analyzed, and at least three leaves of each independent transgenic lines were collected for trichome counting. The quantities of type II trichomes within a 0.5 cm^2^ region and type V and VI trichomes within a 2.2 mm^2^ region were computed.

### GUS staining

β-glucuronidase (GUS) staining was performed as previously described (24). Briefly, *ProSlMYB72*:*GUS* plants were developed by overexpressing the *GUS* gene driven by the *SlMYB72* promoter in the background of WT plants. Tomato leaves from WT and *ProSlMYB72*:*GUS* plants were immersed in GUS staining solution (Coolaber) for 3 h under vacuum conditions and then incubated in the dark for 24 h at 37 °C. Subsequently, the leaves were washed thrice with 75% ethanol and soaked in 75% ethanol for further observation using optical microscopy.

### Yeast two-hybrid assay

A yeast two-hybrid (Y2H) assay was conducted according to the manufacturer’s guidelines (Clontech). Full-length coding regions of *SlMYB72*, *SlMYB75*, and *SlbHLH150* were inserted into pGADT7 vectors, and full-length coding regions of *SlbHLH150* and *SlTTG1* were cloned into pGBKT7 vectors. The resulting pGADT7 and pGBKT7 constructs were co-transformed into Y2HGold yeast cells and selected on a selective dropout (SD) medium lacking tryptophan (Trp) and leucine (Leu). Clones surviving on SD-Trp-Leu plates were further examined for growth on SD medium without Trp, Leu, histidine (His), and adenine (Ade). The primers used in this study are listed in Table S1.

### Biomolecular fluorescence complementation assay

Biomolecular fluorescence complementation (BiFC) assay was performed as previously described (55). Briefly, the open reading frames of *SlMYB72*, *SlMYB75*, and *SlTTG1* were cloned into the pXY106 vector carrying the N-terminus of YFP (nYFP), and the coding regions of *SlbHLH150* and *SlTTG1* were cloned into the pXY104 vector carrying the C-terminus of YFP (cYFP). The resulting constructs were then introduced into the GV3101 strain. *Agrobacterium* cells containing purpose pXY104 and pXY106 were resuspended in infiltration buffer (10 mM MgCl_2_, 200 μM acetosyringone, and 10 mM MES, pH 5.8) and mixed equally. After 2 h of incubation, the cells were co-infiltrated into the leaves of *N. benthamiana*. The transfected plants were maintained in the dark for 24 h and then exposed to light for 48 h. YFP fluorescence emitted from the infiltrated leaves of *N. benthamiana* was observed using a confocal laser scanning microscope (Olympus Fluoview FV1000).

### GST pull-down assay

For the GST (glutathione-S-transferase) pull-down assay, the *SlMYB72*, *SlMYB75*, and *SlTTG1* coding sequences were fused to a GST tag and cloned into the pGEX-4T-1 vector. The coding regions of *SlbHLH150* and *SlTTG1* were cloned into the pCold vector carrying a His tag. The resulting constructs were introduced into BL21(DE3) for recombinant protein expression and purification. Purified His-tagged and GST-tagged recombinant proteins (or GST) were equally mixed and incubated with GST agarose (Yeasen) at 4 °C for 2 h. GST agarose was then collected by centrifugation and washed at least 10 times with phosphate-buffered saline (PBS). Finally, GST agarose was resuspended in an SDS loading buffer and boiled for 10 min. The supernatant was subjected to SDS-PAGE and immunoblotting analysis using primary antibodies against the GST (Proteintech, Cat No 66001-2-1g, 1:5000 dilution, anti-mouse) and His tags (ABclonal, Cat No AE003, 1:5000 dilution, anti-mouse). The secondary antibody was goat anti-mouse IgG HRP (Abmart, M21001, 1:8000 dilution). The signal was generated by BeyoECL Moon kit (Beyotime, D3308-2) and acquired by the chemiluminescence instrument (BioRad, Universal Hodd II Gel Doc XR System).

### Yeast three-hybrid assay

The yeast three-hybrid assay (Y3H) was utilized to assess the competitive relationship between SlMYB72 and SlMYB75 with SlTTG1 and SlbHLH150, and was conducted in accordance with the manufacturer’s instructions (Clontech). The coding sequences of *SlbHLH150* or *SlTTG1* were cloned into the multiple cloning site 1 (MCS1) of the pBridge vector and formed fusion proteins with the GAL4 DNA-binding domain. Meanwhile, the open reading frames of *SlMYB72* or *SlMYB75* were cloned into the MCS2 of the pBridge vector and driven by the *MET25* promoter to express the bridge proteins. The engineered pBridge vectors and the purpose pGADT7 vectors carrying *SlMYB72* or *SlMYB75* were co-introduced into Y2HGold yeast cells and screened on SD-Trp-Leu medium lacking methionine (Met). The growth of viable clones was further evaluated on SD-Trp-Leu-His-Ade medium with or without Met. The transcriptional activity of the *MET25* promoter is activated in the absence of Met, and repressed in the presence of Met.

### Electrophoretic mobility shift assay

The electrophoretic mobility shift assay (EMSA) was performed as previously described (55). Briefly, GST-tagged SlMYB72 (GST-SlMYB72) was expressed in the *E. coli* strain BL21 (DE3) and purified using GST agarose (Yeasen). A fragment of the *SlCycB2* promoter containing the MYB-binding motif was used as a probe and labeled with biotin using a Lightshift Chemiluminescent EMSA Kit (Thermo Fisher Scientific). The unlabeled *SlCycB2* promoter fragment served as a competitor, and the same fragment with a mutated MYB-binding motif (AAAAAA) was used as the mutant probe. The attachment of GST-SlMYB72 to the biotin-labeled probe was determined using a Protein Gel Imager (BIO-RAD).

### Real-time fluorescence quantitative PCR analysis

Total RNA was extracted from the leaves of WT and transgenic plants using the RNeasy Plant Mini Kit (Qiagen, China) and reverse-transcribed using the RevertAid RT Reverse Transcription Kit (Thermo Fisher Scientific), as recommended. Real-time fluorescence quantitative PCR (RT-qPCR) analysis was performed using the All-in-One qPCRMix (GeneCopoeia). The tomato *actin* (*Solyc11g069190*) gene was used as the internal control, and the relative expression levels of the target genes were determined based on the ΔΔCt values. In addition, the relative expression of target genes in wild type were normalized to 1 for comparison with the expression levels of the same target genes in transgenic plants. The primer sequences used for RT-qPCR analysis are listed in Table S1.

### Dual-luciferase reporter assay

The dual-luciferase reporter assay was performed as described previously (52). The promoter region of *SlCycB2* was inserted into the pGreen-0800-LUC vector to drive *luciferase* (*LUC*) gene expression, and the resulting construct was used as a reporter. The coding regions of *SlMYB72*, *SlMYB75*, *SlbHLH150*, and *SlTTG1* were inserted into the pGreen II-62SK vectors, and the targeting constructs were used as effectors. These constructs were introduced into the *Agrobacterium* GV3101 (pSoup) strains. *Agrobacterium* strains containing purpose pGreen II 0800 and pGreen II-62SK were mixed at 1:9 and infiltrated into the leaves of *N. benthamiana*. After 24 h in the dark, leaves were exposed to light for 48 h. The activities of LUC and Renillla (REN) were measured according to the instructions of the dual luciferase assay kit (Promega) using a microplate reader (Biobase, China). *SlCycB2* promoter activity was assessed by calculating the relative ratio of LUC and REN.

### Genetic hybridization

Different unflowering OE-*SlMYB75* and RNAi-*SlMYB75* plants, which were demasculinized and retained stigmas, were selected as the parents. Different already-flowering OE-*SlMYB72* and RNAi-*SlMYB72* plants were also selected as parents. Anthers from the aforementioned already-flowering plants were scraped from the petals using forceps and affixed to the stigma of the demasculinized parent. The hybrid plants were cultivated in a growth chamber until the fruit matured. Tomato seeds were harvested from the hybrid fruits and planted for further identification.

### Statistical analysis

Experiments were conducted with a minimum of three replications. Significant differences between the control and experimental groups were assessed using univariate ANOVA and Tukey’s honestly significant difference test. Differences were considered statistically significant at *p* < 0.05.

## Data availability

All data supporting this study are contained within the manuscript and supplemental information.

## Supporting information

This study contains five supplemental figures (Figs. S1–S5) and a supplemental table (Table S1) uploaded as Excel file. This article contains supporting information.

## Conflict of interest

The authors declare that they have no conflicts of interest with the contents of this article.
